# Human Herpesvirus 1 in Wild Marmosets, Brazil, 2008

**DOI:** 10.3201/eid1707.100333

**Published:** 2011-07

**Authors:** Camila S. Longa, Sávio F. Bruno, Amaury R. Pires, Phyllis C. Romijn, Leda S. Kimura, Carlos H.C. Costa

**Affiliations:** Author affiliations: Empresa de Pesquisa Agropecuária do Estado de Rio de Janeiro, Niterói, Rio de Janeiro, Brazil (C.S. Longa, P.C. Romijn, L.S. Kimura, C.H.C. Costa);; Universidade Federal Fluminense, Niterói, Rio de Janeiro (C.S. Longa, S.F. Bruno);; Secretaria de Agricultura, Pecuária, Pesca e Abastecimento do Estado do Rio de Janeiro, Niterói, Rio de Janeiro (A.R. Pires)

**Keywords:** viruses, Herpesviridae infections, human herpesvirus 1, zoonoses, New World monkeys, primates, viruses, Brazil, letter

**To the Editor**: Human herpesvirus 1 (HHV-1) infections in New World monkey species, especially in the Callithrichid family, have been described (*1*–*6*), but most reports have discussed experimental infections or isolated spontaneous infections in pet, zoo, or research animals. We report an outbreak of HHV-1 in wild marmosets (*Callithrix* spp.) in the city of Rio de Janeiro, Brazil.

In October 2008, the Empresa de Pesquisa Agropecuária received 5 marmosets (*Callithrix* spp.) from the Campo Grande district of Rio de Janeiro for necropsy. These animals were usually fed by residents of a condominium complex and were having neurologic signs and severe prostration, physiologic changes suggestive of herpesvirus infections. Euthanasia, followed by necropsy and histopathologic examinations to determine the cause of illness, were recommended.

The primary changes observed during necropsy were vesicular and necrotic plaques on tongues (Figure A1, panel A) and ulcerations in oral mucosa of all examined animals, as well as large lymph nodes of the cervical region, mainly retropharyngeal. Three animals showed marked brain congestion (Figure A1, panel B). Other alterations were splenomegaly, lung congestion, and adrenomegaly.

Histopathogic examinations found superficial ulcerations of the tongue, variable in dimension, that showed fibrinopurulent exudates, mononuclear cell infiltrates on lamina propria, and balloon degeneration of epithelial cells. The brains had multifocal nonsuppurative meningoencephalitis with perivascular and vascular infiltrates of mononuclear cells and gliose foci (Figure, panels A, B). Adrenal glands had hyperemia, hemorrhage, perivascular infiltrates of mononuclear cells, and focal necrosis. Mild hyperemia and alveolar emphysema had occurred in lungs. The livers showed hyperemia and mild to moderate periportal infiltrates of mononuclear cells. Lymph nodes showed hemorrhages, lymphoid hyperplasia, and small foci of subcapsular necrosis. Hyperemia and decreased lymphoid cells population were present in the spleens. In addition, intranuclear inclusion bodies in cells of brains, peripherical nerves, tongues, and adrenal glands were observed. These changes were found in all animals. All changes were consistent with HHV-1 in nonhuman primates (*2*–*8*).

To confirm the diagnosis, immunohistochemical examination was done by using polyclonal antibody directed against HHV-1. We used the avidin–biotin–peroxidase complex method with Harris hematoxylin counterstain. Sections taken of the ulcerated oral lesions had intranuclear inclusion areas strongly marked by immunoperoxidase (Figure, panels C, D). HHV-1 infection was confirmed in the 5 marmosets.

Many reports have described human herpesvirus in New World monkeys. Most of the reports were of experimental or isolated spontaneous infections in pets (*1**,**2*), zoo (*3*), research (*4**,**5*) or wild animals (*6*). This is the second report of a naturally occurring infection in wild marmosets. Both infections occurred in the Grande Rio region, where *Callithrix* spp. imported from other Brazilian states were accidentally introduced. These species came to occupy a niche that once belonged to the golden lion tamarin (*Leontopitecus rosalia*) (*9**,**10*).

Humans are the reservoir and the natural host of human herpesvirus (*3**–**6*), which can be disseminated by direct contact, through sexual activity (*5*) and, in a brief period after contamination, through domestic tools and food remains (*6*). Once brought to the colony, the disease spreads quickly with high rates of illness and death (*4**,**5*). In general, the herpesviruses produce asymptomatic and latent infections in their natural hosts but cause severe disease when transmitted to other species (*5**,**7**,**8*).

In Old World primates, benign and localized human herpesvirus infections have been described. Although systemic infections with fatal outcome occur, infection usually remain confined to the skin, oral cavity, external genitalia, and conjunctiva (*1**–**3**,**5**,**6*) rather than affecting the nervous system.

New World primates are highly susceptible to infection and severe disease, with spontaneous infections more commonly reported in *Callithrix* spp. The clinical course is severe, resulting in death in most reported cases (*2**,**4**,**5*). In marmosets, human herpesvirus produces an epizootic disease with substantial illness and death (*7*). This viral infection has already been described in 3 species of marmosets (*C. jacchus, C. penicillata* and *C. geoffroyi*) and in owl monkeys (*Aotus trivirgatus*) and cotton-head tamarins (*Saguinus oedipus*) (*1**–**3**,**5*).

There is only 1 report of spontaneous infection in free-living black tufted-ear marmosets (*C. penicillata*), which occurred at the State Park of Serra da Tiririca, Niterói, Brazil (*6*). In this report, the infection is thought to have been related to the proximity between local human residents and wildlife; the disease also reportedly developed with substantial illness and death in the marmoset population (*6*). Similarly, the cases presented here presumably were acquired from close contact with humans because the animals were fed regularly at a residential condominium, and the virus can be transmitted through contact with contaminated saliva, aerosols, and fomites, such as tools. The high susceptibility and mortality rates for New World monkeys that contract this infection argues strongly for prophylactic strategies, considering that the infection occurs even in conservation parks and could seriously affect the local primatologic fauna and thus species conservation.

**Figure:**
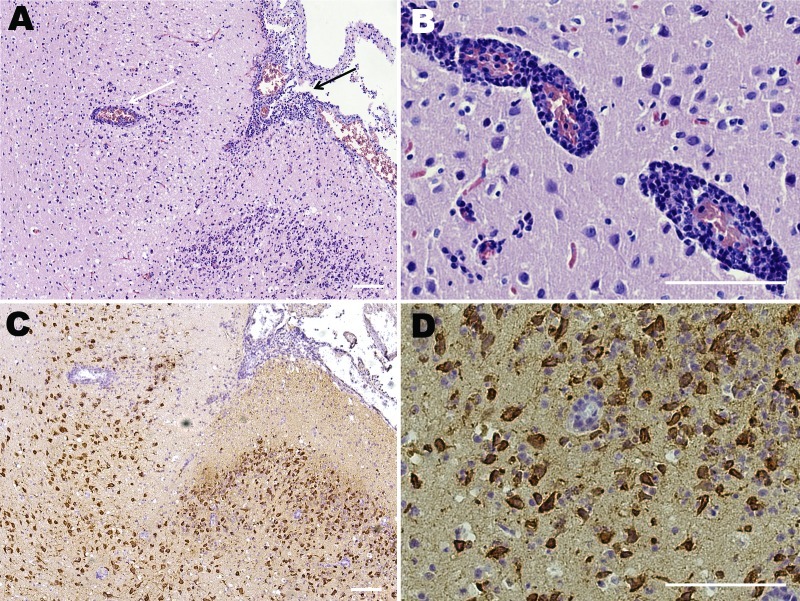
Microscopic lesions of brain caused by human herpesvirus 1 infection in marmosets. A) Histopathogic sample stained with hematoxylin and eosin showing nonsuppurative meningoencephalitis with perivascular infiltrates (black arrow) and infiltrates in piamater (white arrow). B) Histopathologic sample stained with hematoxylin and eosin showing perivascular and vascular infiltrates of mononuclear cells. C, D) Immunohistochemical examination by using polyclonal antibody directed against human herpesvirus 1 and the avidin–biotin–peroxidase complex method, Harris hematoxylin counterstain. Neural cells strongly marked by immunoperoxidase, indicating a positive finding. Scale bars = 100 μm.

## Figures and Tables

**Figure A1:**
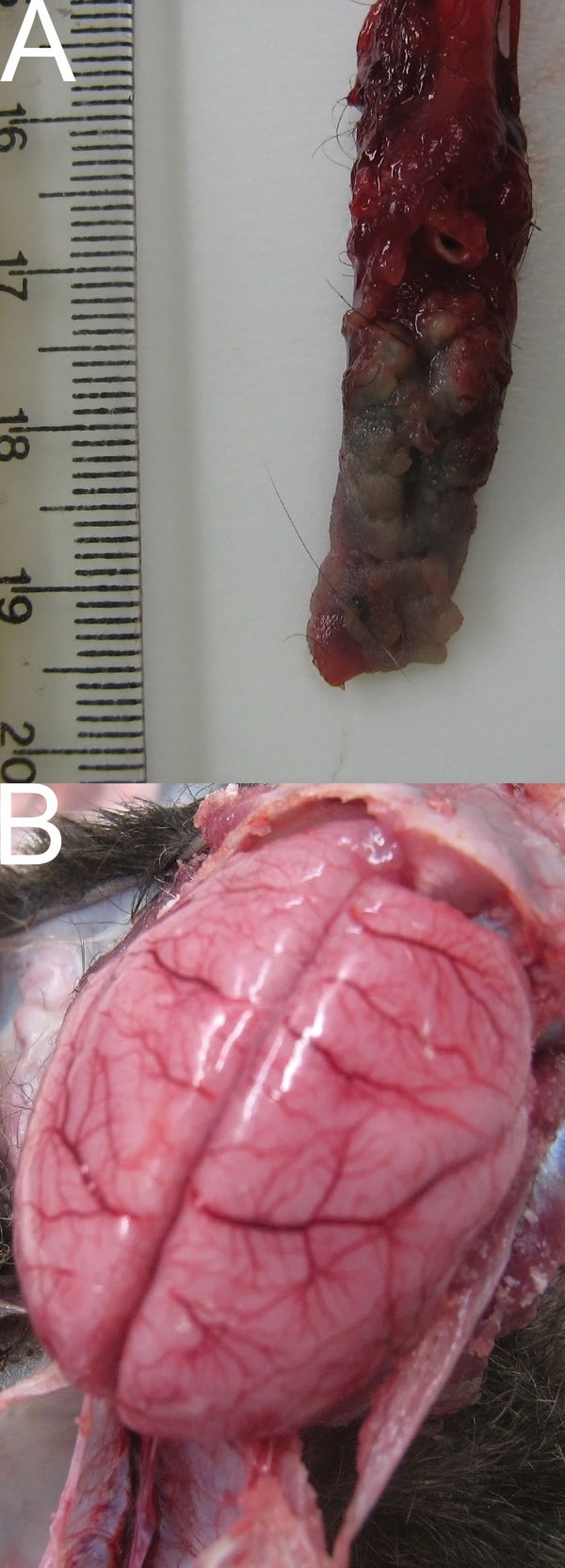
Gross lesions caused by human herpesvirus 1 infection in marmosets. A) Vesicular and necrotic plaques in tongue. B) Marked brain congestion. Scale shown in centimeters.
